# Supplementary material for: Identification of serum metabolites associating with chronic kidney disease progression and anti-fibrotic effect of 5-methoxytryptophan
Source: Nat Commun. 2019 Apr 1;10:1476. doi: 10.1038/s41467-019-09329-0 (PMC6443780; doi:10.1038/s41467-019-09329-0)
Supplement: Supplementary file 1 — Supplementary Information [file 41467_2019_9329_MOESM1_ESM.pdf]

**Identification of serum metabolites associating with chronic kidney disease progression and anti-fibrotic effect of 5-methoxytryptophan**

Chen *et al*.

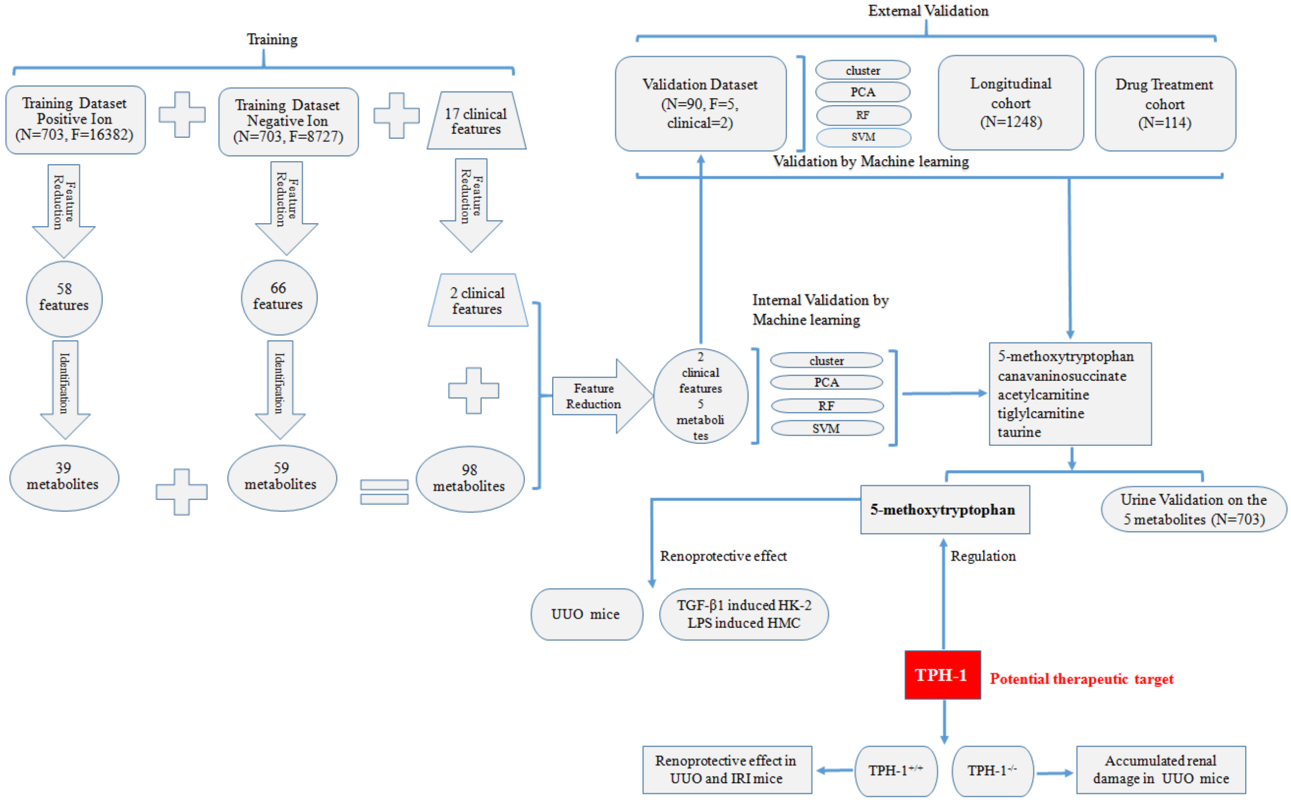


**Supplementary Figure 1. The overview of study design.** Flow diagram of metabolomic analysis presented the phases of biomarker discover and biomarker validation by a cohort of 90 healthy controls and patients with stages 1-2 CKD; a longitudinal cohort study of 1,248 patients without CKD followed for up to six years; and a pre- and post-drug intervention study of 114 patients with CKD2. Five metabolites, 5-MTP, CSA, acetylcarnitine, tiglylcarnitine and taurine were identified as biomarkers for progressive CKD. 5-MTP was selected for further investigation. 5-MTP and its regulatory TPH-1 enzyme attenuated renal inflammation and fibrosis to reduce renal injury. TPH-1 has the potential to be developed as a new target for new drug discovery and treatment of CKD. RF, Random Forest; SVM, Support vector machine.

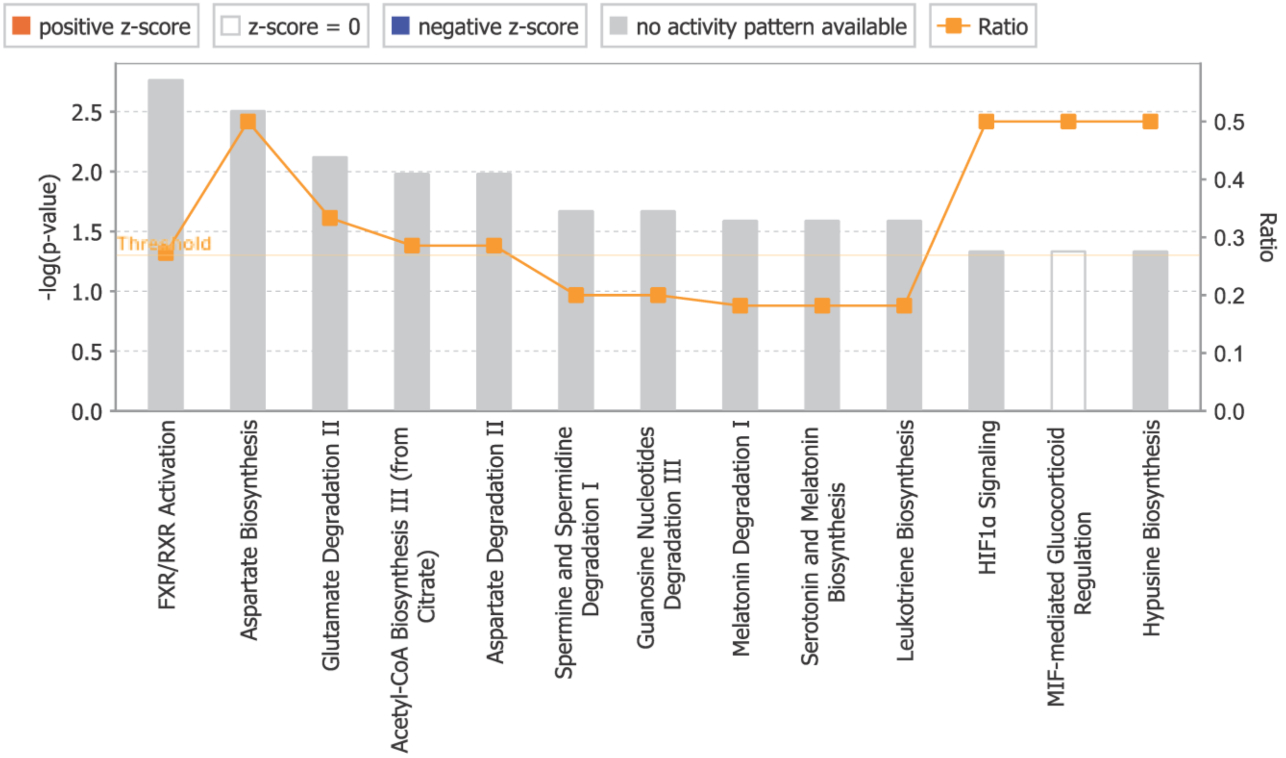


**Supplementary Figure 2. The significant Canonical Pathways identified with the 98 metabolites.** The pathways are displayed along the x-axis. As the default the y-axis displays the -log of p-value which is calculated by right trailed Fisher's exact test. Thus taller bars equate to increased significance and the canonical pathways are sorted by significance from left to right. White bars are those with a z-score at or very close to 0. Gray bars indicate pathways where no prediction can currently be made. The orange points connected by a thin line represent the Ratio. The ratio is calculated as follows: # of genes in a given pathway that meet your cutoff criteria, divided by the total # of metabolites that make up that pathway and that are in the reference gene set.

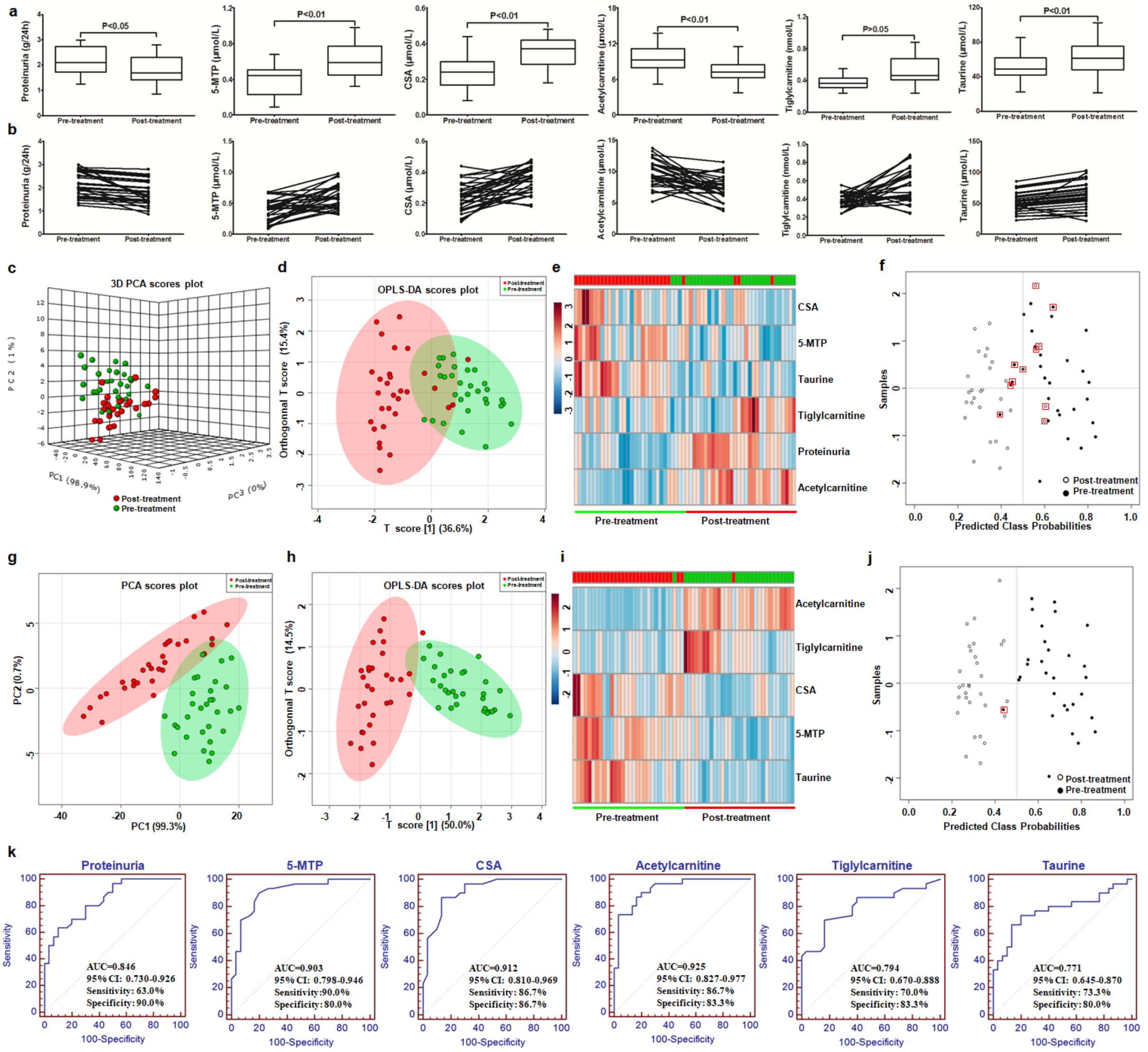


**Supplementary Figure 3. Five potential biomarkers were further validated in pre- and post-treatment by prednisone.** **a** Box plots of levels of five potential biomarkers including 5-MTP, CSA, acetylcarnitine, tiglylcarnitine and taurine in patients with CKD2 pre- and post-treatment by prednisone. Mean values are presented by horizontal bars. The whiskers indicate the maximum and minimum points. **b** Dots and lines showing changes of five potential biomarkers in each individual from patients with CKD2 pre- and post-treatment by prednisone. **c** PCA of two components of proteinuria and five biomarkers. **d** OPLS-DA of two components of proteinuria and five biomarkers. **e** Heatmap of two components of proteinuria and five biomarkers. **f** Diagnostic performances of two components of proteinuria and five biomarkers based on the PLS-DA model. The black dots with red squares or black circles with blue squares are for the incorrectly predicted samples in pre- and post-treatment. 25 Out of the 30 patients with CKD2 of pre-treatment were located in pre-treatment area (83.3% specificity) and 24 out of the 30 patients with CKD2 of post-treatment were correctly grouped (80.0% sensitivity). **g** PCA of two components of five biomarkers. **h** OPLS-DA of five biomarkers. **i** Heatmap of five biomarkers. **j** Diagnostic performances of five biomarkers based on the PLS-DA model. The black dots with red squares and or black circles with blue squares are for the incorrectly predicted samples in pre- and post-treatment. 29 Out of the 30 patients with CKD2 of pre-treatment were located in pre-treatment area (96.7% specificity) and all the 30 patients with CKD2 of post-treatment were correctly grouped (100.0% sensitivity). These results demonstrated that the five biomarkers show high prediction class probabilities. **k** Analysis of PLS-DA based ROC curves of proteinuria and five potential biomarkers in patients with CKD2 pre- and post-treatment by prednisone. The associated AUC, 95% confidence interval (CI), sensitivity and specificity values were indicated. Student's *t*-test was used for the significance of difference between two groups.

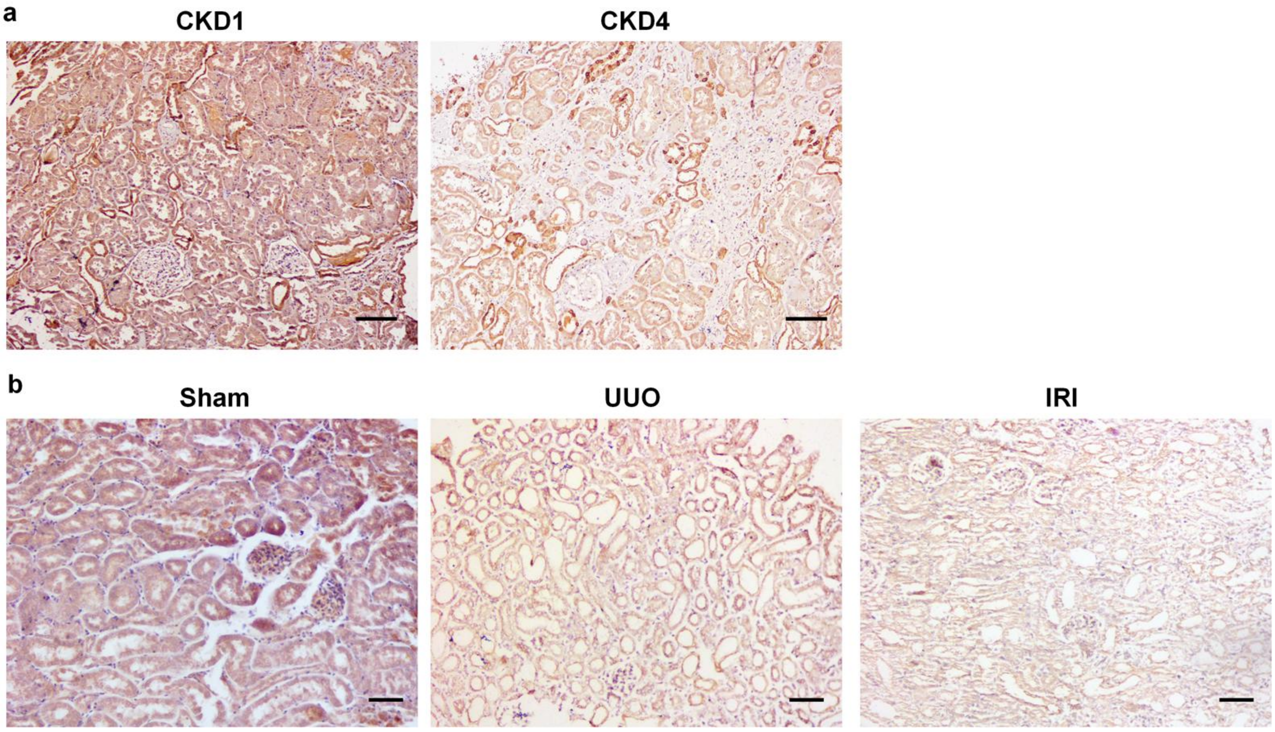


**Supplementary Figure 4. TPH-1 staining of kidney tissues. a** TPH-1 staining of kidney tissues of patients with CKD1 and CKD4, respectively. Scale bar 400 μm. **b** TPH-1 staining of kidney tissues of UUO and IRI mice. In normal station. Scale bar 50 μm.

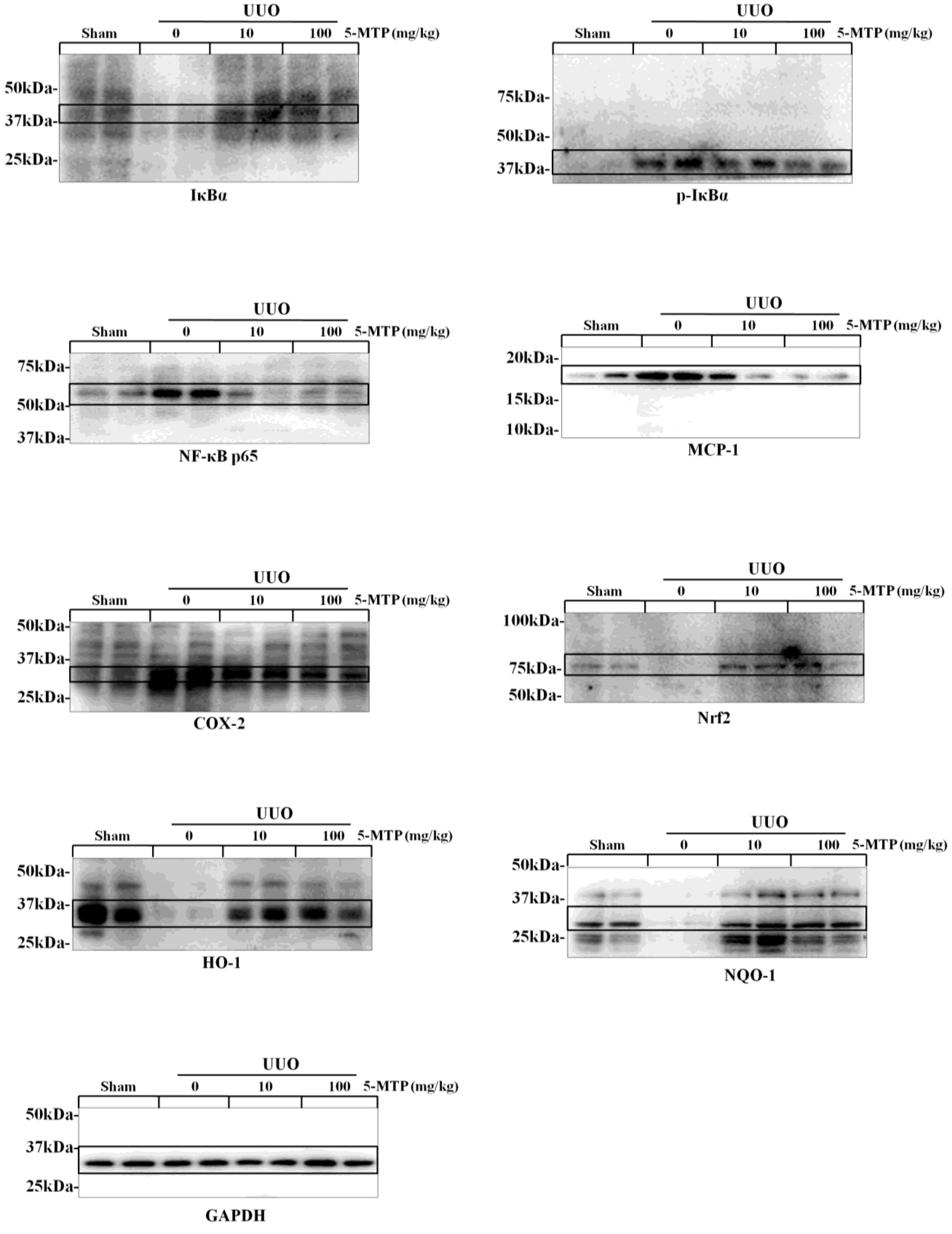


**Supplementary Figure 5. Detailed western blots for Figure 6c.** The relevant figures are indicated in the blots.

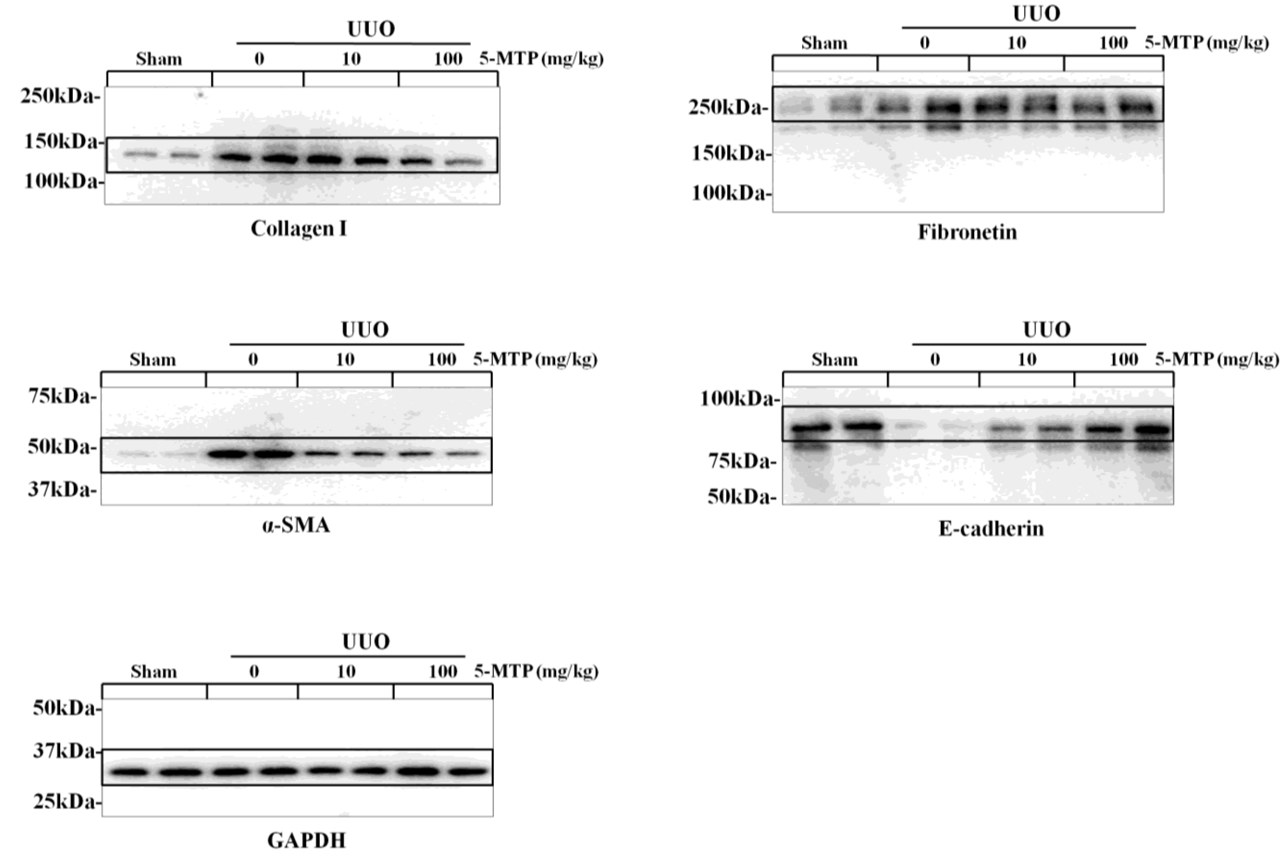


**Supplementary Figure 6. Detailed western blots for Figure 6h.** The relevant figures are indicated in the blots.

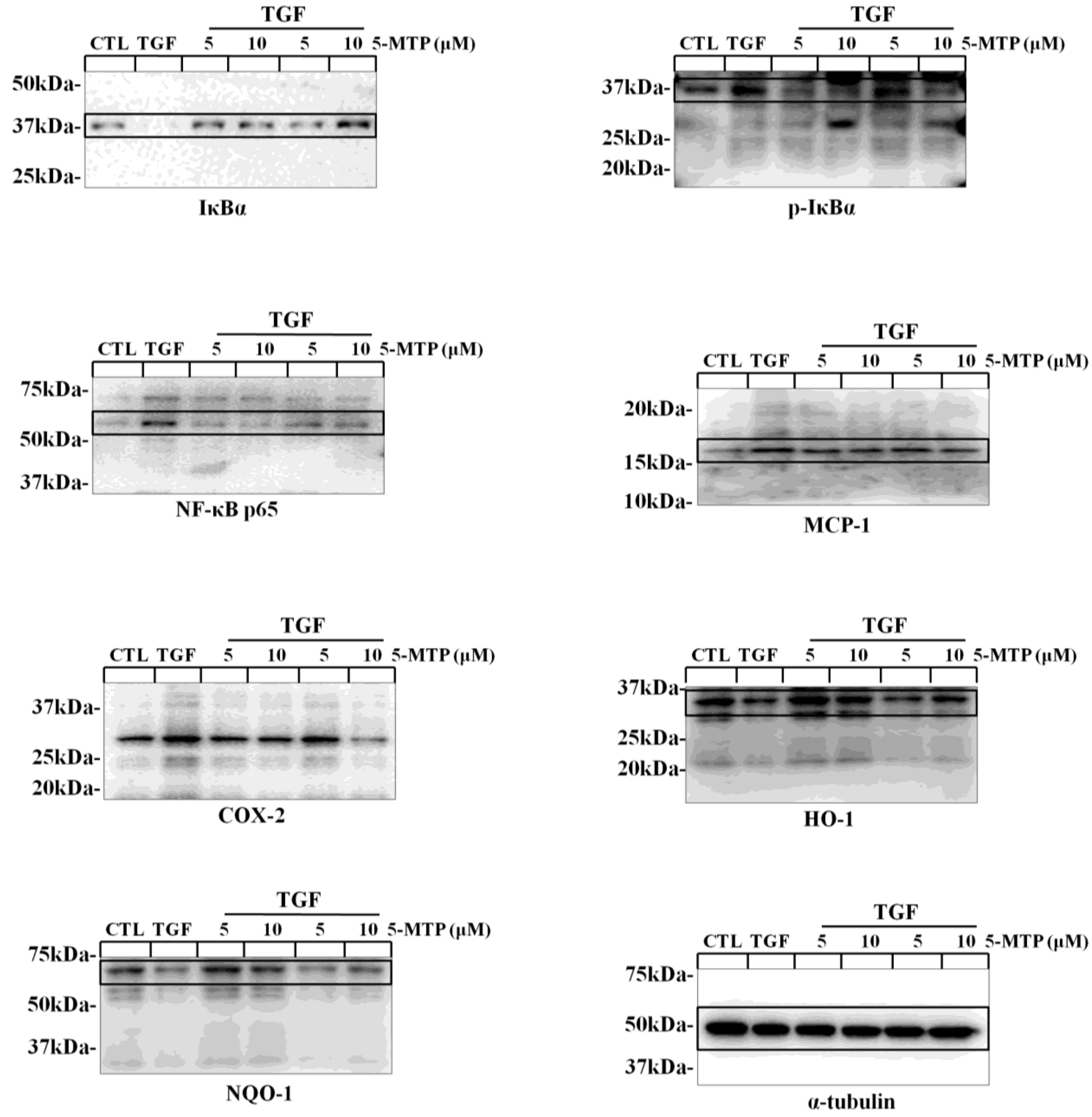


**Supplementary Figure 7. Detailed western blots for Figure 7a.** The relevant figures are indicated in the blots.

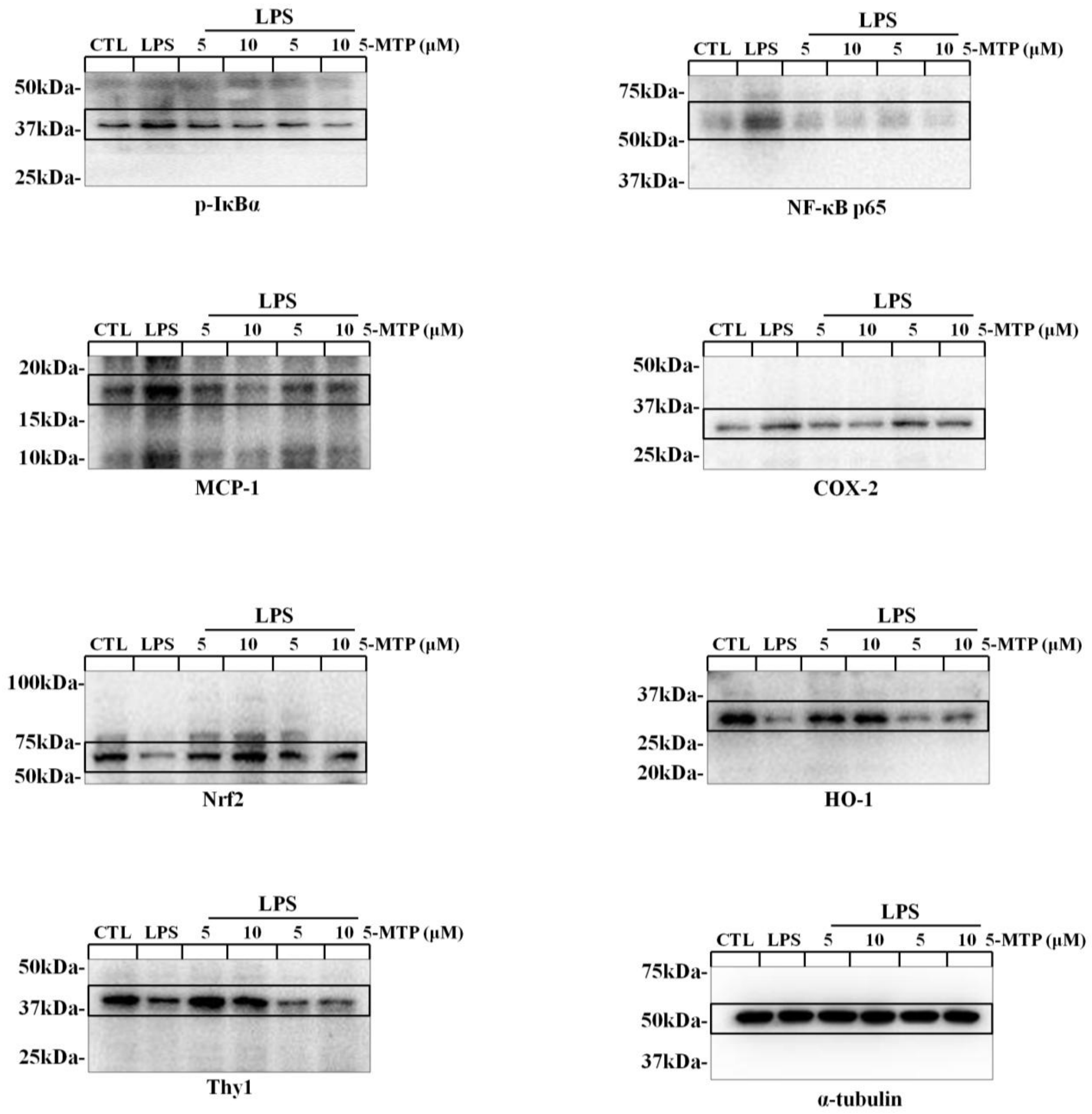


**Supplementary Figure 8. Detailed western blots for Figure 7d.** The relevant figures are indicated in the blots.

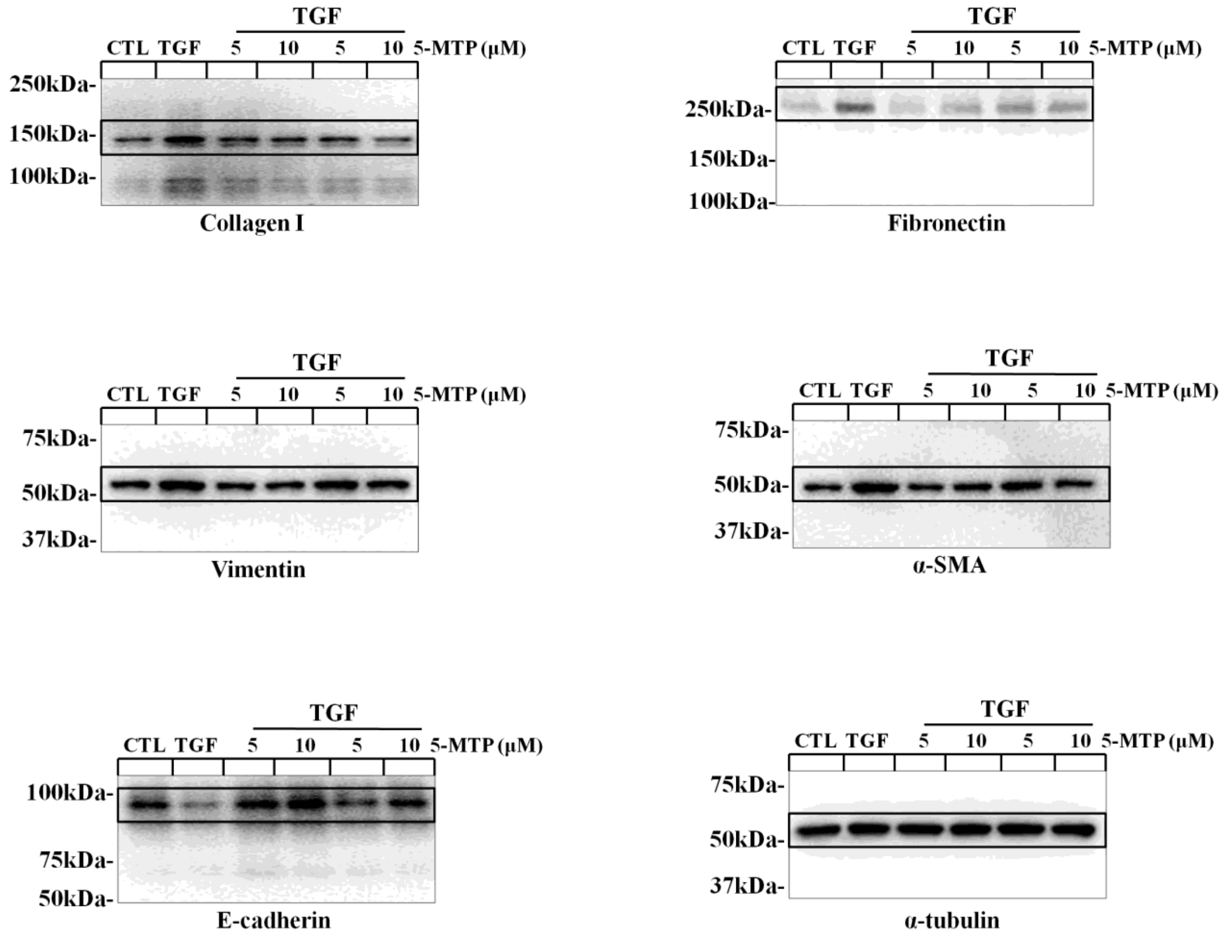


**Supplementary Figure 9. Detailed western blots for Figure 7g.** The relevant figures are indicated in the blots.

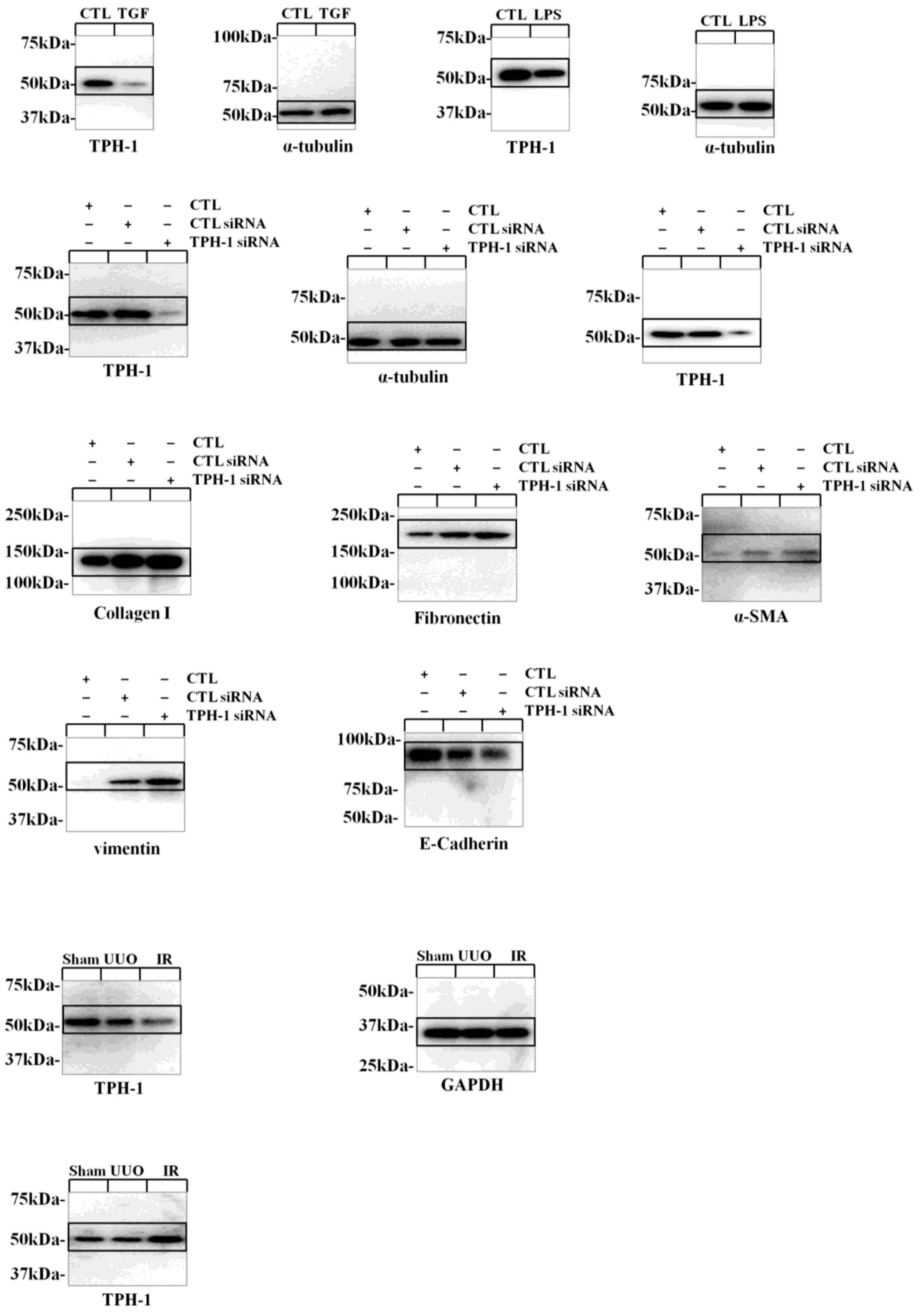


**Supplementary Figure 10. Detailed western blots for Figure 8a-c, e and f.** The relevant figures are indicated in the blots.

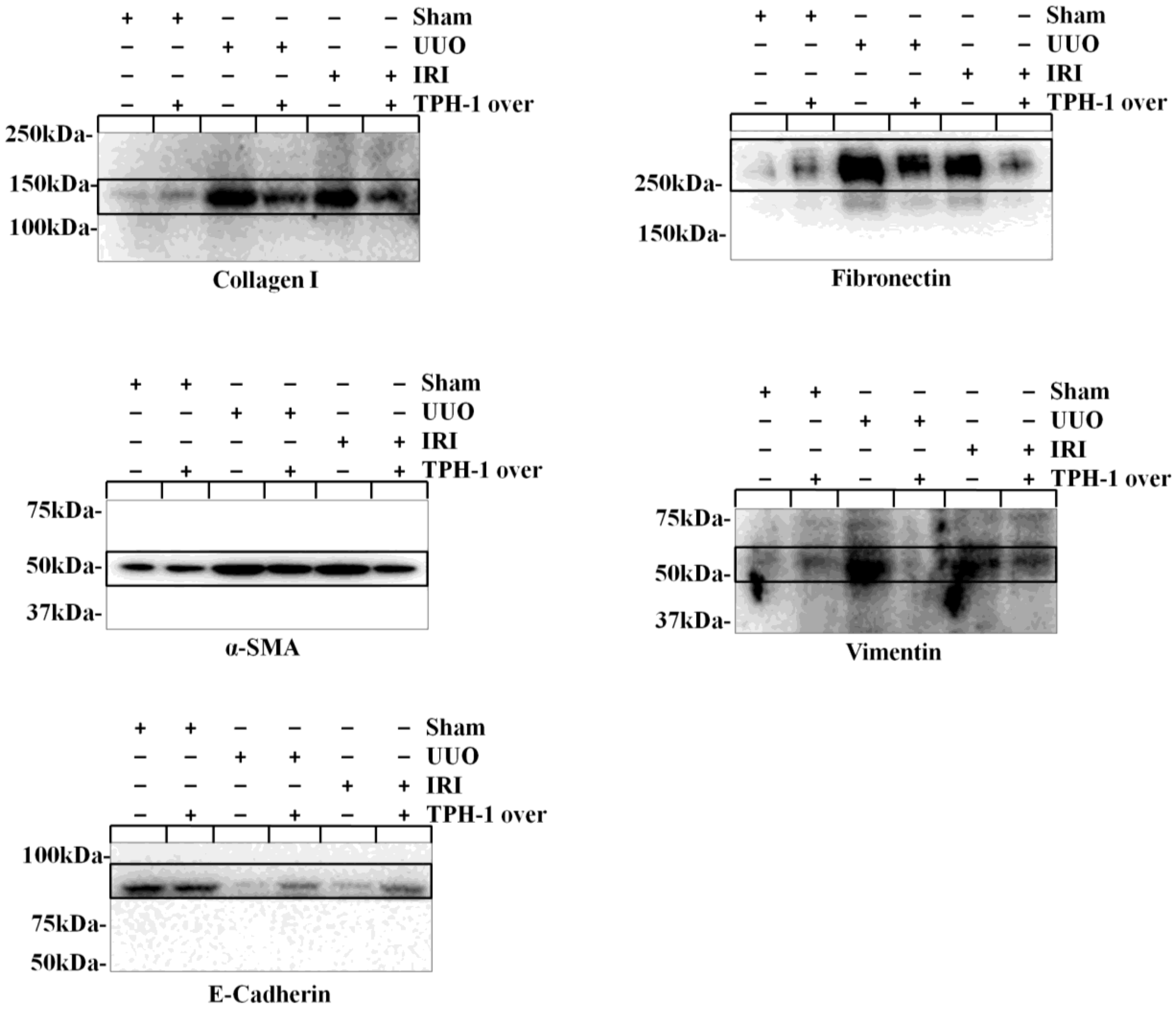


**Supplementary Figure 11. Detailed western blots for Figure 8h.** The relevant figures are indicated in the blots.

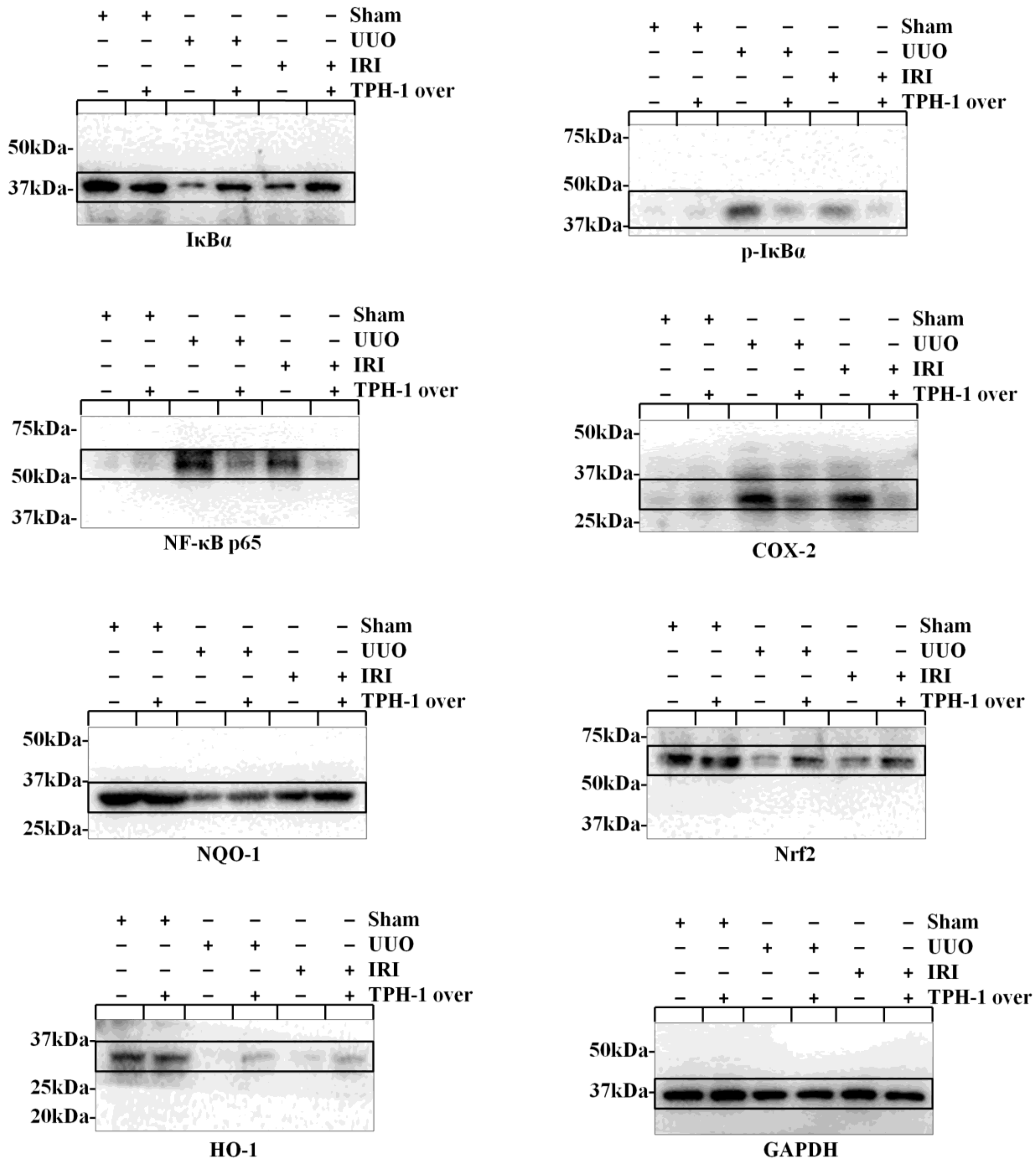


**Supplementary Figure 12. Detailed western blots for Figure 8j.** The relevant figures are indicated in the blots.

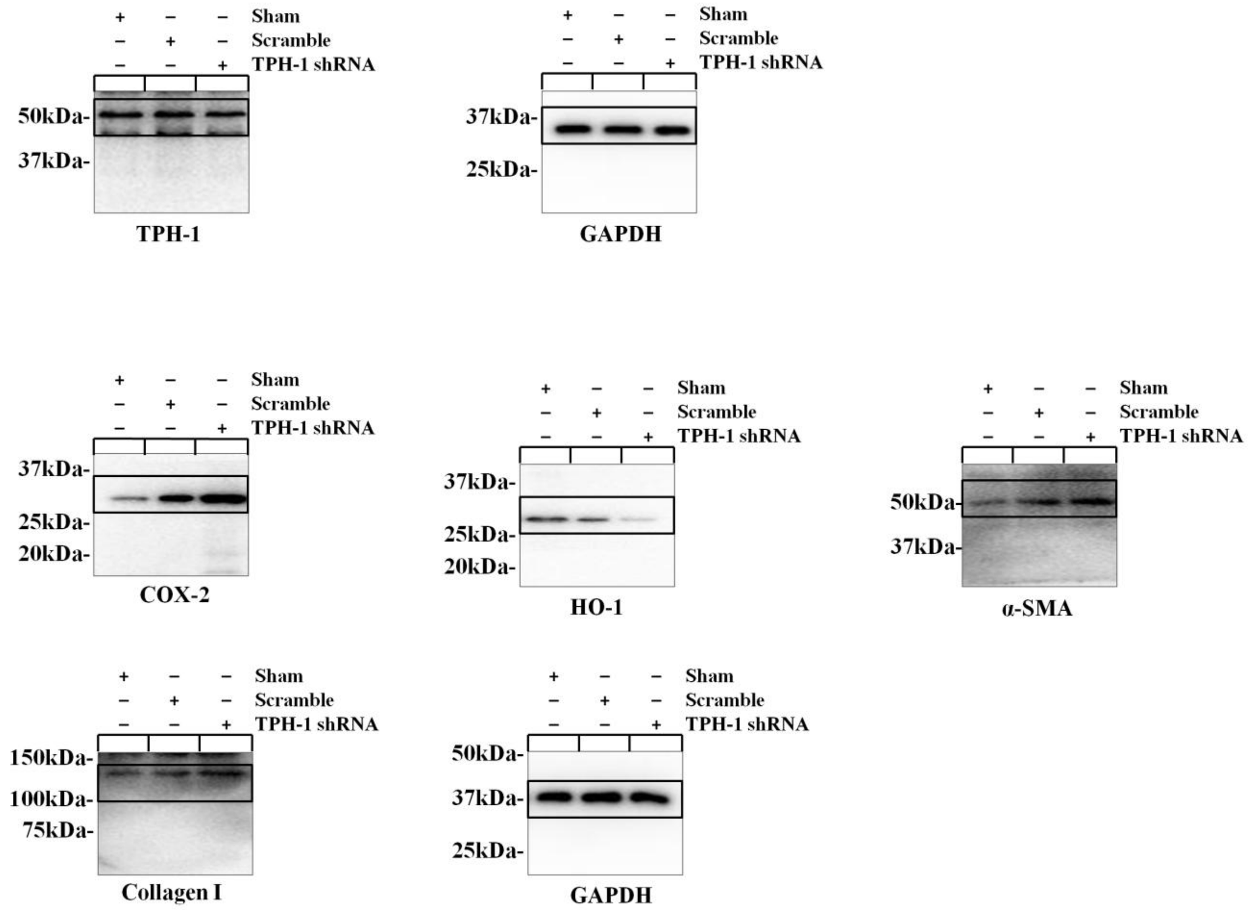


**Supplementary Figure 13. Detailed western blots for Figure 8l and n.** The relevant figures are indicated in the blots.

**Supplementary Tables**

**Supplementary Table 1 Clinical and demographic baseline characteristics of healthy controls and CKD patients**

| Clinical characteristics | Normal | CKD1 | CKD2 | CKD3 | CKD4 | CKD5 | Effect[a] | P[b] |
|---|---|---|---|---|---|---|---|---|
| Sample size | 116 | 120 | 104 | 110 | 119 | 134 | NA | NA |
| Men (%) | 56.8 | 49.1 | 48.1 | 68.1 | 52.1 | 47 | NA | NA |
| eGFR (mL/min/1.73 $m^2$) | 103.5±12.9 | 104.9±13.3 | 75.4±8.8** | 43.9±12.3** | 21.0±4.5** | 8.9±4.5** | NA | NA |
| Age (years) | 54±14 | 53±16 | 52±11 | 54±15 | 60±15** | 58±15* | 0.0104 | 3.31E-02 |
| BW (kg) | 68±11 | 69±12 | 72±12** | 78±13** | 79±11** | 80±13** | 0.012 | 1.26E-04 |
| BMI ($kg/m^2$) | 23.7±3.7 | 24.7±4.1 | 24.9±5.2 | 26.4±4.4** | 27.3±4.1** | 27.9±5.1** | 0.0519 | 1.24E-06 |
| SBP (mm Hg) | 121±17 | 124±18 | 125±16* | 140±27** | 138±20** | 144±24** | 0.0009 | 1.15E-01 |
| DBP (mm Hg) | 76±12 | 77±12 | 79±12* | 84±17** | 75±12 | 80±16** | -0.0023 | 3.94E-01 |
| Total protein (g/L) | 62.5±3.3 | 51.0±12.4** | 61.9±11.8 | 66.2±10.1** | 61.7±8.9 | 60.5±9.0 | 0.0271 | 1.55E-06 |
| Albumin (g/L) | 42.0±3.7 | 32.1±8.2** | 36.2±8.7** | 38.0±6.6** | 36.1±6.1** | 35.4±6.2** | -0.0513 | 7.62E-11 |
| TC (mmol/L) | 4.51±1.30 | 4.95±2.64** | 4.30±1.95 | 4.61±1.52 | 4.50±1.49 | 4.30±1.26 | -0.0578 | 1.50E-02 |
| TG (mmol/L) | 1.83±1.45 | 2.03±1.28 | 2.11±1.56 | 1.84±1.30 | 1.71±1.35 | 1.43±0.90 | -0.0312 | 2.63E-01 |
| HDL-C (mmol/L) | 1.77±0.62 | 1.72±0.49 | 1.70±0.49 | 1.67±0.48 | 1.64±0.46* | 1.59±0.47** | 0.0394 | 5.82E-01 |
| LDL-C (mmol/L) | 2.90±1.00 | 4.48±2.02** | 3.22±1.53* | 3.65±1.68** | 3.72±1.76** | 3.72±1.77** | -0.0205 | 4.70E-01 |
| CREA (μmol/L) | 68±13 | 58±16 | 92±18** | 153±33** | 256±60** | 598±280** | 0.0025 | 8.71E-24 |
| Urea (mmol/L) | 5.5±5.4 | 4.8±1.4 | 5.9±2.1 | 9.2±2.5** | 15.9±6.6** | 22.3±8.0** | 0.0697 | 2.12E-18 |
| Uric acid (μmol/L) | 327±93 | 323±100 | 315±76 | 437±123** | 418±111** | 423±138** | 0.0008 | 1.35E-02 |
| P/C ratio | 0.15±0.04 | 3.94±3.28** | 2.14±1.89** | 2.53±2.19** | 2.70±2.38** | 2.76±2.41** | 0.1008 | 3.06E-05 |
| Proteinuria (g/24h) | 0.07±0.02 | 2.84±2.31** | 2.29±2.23** | 2.26±2.36** | 2.17±2.07** | 2.30±2.25** | 0.0367 | 1.90E-01 |

Associations of clinical variables with CKD stages. Results are expressed as the means ± SD, $^{*}P<0.05$, $^{**}P<0.01$ compared with healthy controls. "NA", not applicable. P/C, urine protein/urine creatinine; SPB, systolic pressure blood; DPB, diastolic pressure. [a] Positive effect indicates that the clinical variable is positively associated with CKD stage; negative effect indicates that the clinical variable is negatively associated with CKD stage. [b] P value was computed using original regression.

**Supplementary Table 2 Final model with 5 metabolites and 2 clinical indexes in patients with CKD five stages**

| Metabolites | Effect[a] | Std Err[b] | P[c] |
|---|---|---|---|
| 5-Methoxytryptophan | -0.0111 | 0.0015 | 8.22E-13 |
| Canavaninosuccinate | -0.0069 | 0.0016 | 2.99E-05 |
| Acetylcarnitine | 0.0072 | 0.0010 | 3.19E-13 |
| Tiglylcarnitine | -0.0109 | 0.0019 | 7.17E-09 |
| Taurine | -0.0058 | 0.0011 | 1.63E-07 |
| CREA | 0.0010 | 0.0001 | 4.81E-16 |
| Urea | 0.0197 | 0.0036 | 5.60E-08 |

Model adjusted $R^2$ =94.1%. [a] Positive effect denotes positive association between metabolite/clinical variable and CKD stage; negative effect denotes negative association between metabolite/clinical variable and CKD stage. [b] Std Err, standard error. Std Err was computed from ordinal regression. [c] P values were computed from ordinal regression.

**Supplementary Table 3 Clinical and demographic baseline characteristics of healthy controls and CKD patients**

| Variable | Healthy controls | CKD1 | CKD2 |
|---|---|---|---|
| Sample size | 30 | 30 | 30 |
| Men (%) | 53.3 | 56.3 | 50.0 |
| Age (years) | 56 ±15 | 58 ±18 | 57 ±16 |
| BMI (kg/m$^2$) | 25.1 | 25.5 | 26.4 |
| Hypertension (%) | 21 | 60 | 63 |
| eGFR (mL/min/1.73 m$^2$) | 98.2 ±13.1 | 99.3 ±14.6 | 68.7 ±12.8$^{**}$ |
| CREA (μmol/L) | 67.3 ±15.2 | 65.7 ±13.2 | 85.4 ±19.1$^{**}$ |
| Urea (mmol/L) | 5.3 ±2.1 | 5.4 ±1.7 | 6.2 ±2.8 |
| Proteinuria (g/24h) | N/A | 1.9 ±1.3$^{**}$ | 2.1 ±1.6$^{**}$ |

Results are expressed as the number (%) or means ± SD, $^{*}P<0.05$, $^{**}P<0.01$ compared with healthy controls. N/A, not applicable. One-way ANOVA followed by Dunnett's *post hoc* test for multiple comparisons was used for three or more groups.

**Supplementary Table 4 Association between clinical indexes and eGFR**

| feature | Effect[a] | Std Err[b] | P[c] |
|---|---|---|---|
| 5−Methoxytryptophan | 0.0036 | 0.0006 | 0.0000 |
| Canavaninosuccinate | 0.0004 | 0.0001 | 0.0000 |
| Acetylcarnitine | -0.0030 | 0.0003 | 0.0000 |
| Tiglylcarnitine | 0.0004 | 0.0001 | 0.0000 |
| Taurine | 0.0004 | 0.0001 | 0.0000 |
| CREA | -0.2119 | 0.0761 | 0.0066 |
| Urea | -1.1237 | 0.8026 | 0.1650 |
| Sex | 1.8412 | 2.9076 | 0.5282 |
| Age | -0.1648 | 0.0876 | 0.0632 |
| BW (kg) | -0.0602 | 0.1375 | 0.6625 |
| BMI ($kg/m^2$) | -2.4467 | 0.6538 | 0.0003 |
| SBP (mm Hg) | -0.0675 | 0.0852 | 0.4308 |
| DBP (mm Hg) | -0.2064 | 0.1323 | 0.1222 |
| Total protein (g/L) | -0.0580 | 0.1397 | 0.6791 |
| Albumin (g/L) | 0.2700 | 0.1499 | 0.0752 |
| TC (mmol/L) | 0.6270 | 0.6248 | 0.3184 |
| TG (mmol/L) | -1.1422 | 0.8643 | 0.1897 |
| HDL-C (mmol/L) | -1.5051 | 1.1176 | 0.1815 |
| LDL-C (mmol/L) | 0.5331 | 0.6888 | 0.4410 |
| Uric acid (μmol/L) | -0.0172 | 0.0152 | 0.2633 |
| P/C ratio | -22.0000 | 6.6573 | 0.0037 |
| Proteinuria (g/24h) | -1.3152 | 0.6314 | 0.0402 |

[a] Positive effect denotes positive association between metabolite/clinical variable and eGFR. Negative effect denotes negative association between metabolite/clinical variable and eGFR. [b] Std Err, standard error. Std Err was computed from ordinal regression. [c] P values were computed from ordinal regression.

**Supplementary Table 5 Baseline characteristics of the longitudinal cohort study sample**

| Clinical Characteristics | Participants who developed CKD | Participants who did not develop CKD |
|---|---|---|
| Sample size | 31 | 1217 |
| Age (years) | 59.5 ±7** | 55.8 ±8 |
| Men (%) | 54 | 52 |
| BMI(kg/$m^2$) | 25.4 | 25.8 |
| Diabetes (%) | 12** | 4 |
| Hypertension (%) | 67** | 37 |
| Dipstick proteinuria (%) | 42** | 29 |
| eGFR (mL/min/1.73 $m^2$) | 87.5 ±22.7* | 96.3 ±24.4 |
| eGFR at follow-up (mL/min/1.73 $m^2$) | 50.2 ±9.4** | 88.7 ±20.4 |

Results are expressed as the number (%) or mean ± SD, *$P$<0.05, **$P$<0.01 compared with participants who did not develop.
