# Supplementary material for: Identification of serum metabolites associating with chronic kidney disease progression and anti-fibrotic effect of 5-methoxytryptophan
Source: Nat Commun. 2019 Apr 1;10:1476. doi: 10.1038/s41467-019-09329-0 (PMC6443780; doi:10.1038/s41467-019-09329-0)
Supplement: Supplementary file 3 — Description of Additional Supplementary Files [file 41467_2019_9329_MOESM3_ESM.docx]

**Description of Supplementary Files**

**File Name:** Supplementary Data 1

**Description:** Univariate model fitting the 98 metabolites selected by LASSO regression.
