# Supplementary material for: Identification of serum metabolites associating with chronic kidney disease progression and anti-fibrotic effect of 5-methoxytryptophan
Source: Nat Commun. 2019 Apr 1;10:1476. doi: 10.1038/s41467-019-09329-0 (PMC6443780; doi:10.1038/s41467-019-09329-0)
Supplement: Supplementary file 5 — Reporting Summary [file 41467_2019_9329_MOESM5_ESM.pdf]

nature research

Corresponding author(s): NCOMMS-18-11249B

Last updated by author(s): Feb 19, 2019

Reporting Summary

Nature Research wishes to improve the reproducibility of the work that we publish. This form provides structure for consistency and transparency in reporting. For further information on Nature Research policies, see Authors & Referees and the Editorial Policy Checklist.

Statistics

For all statistical analyses, confirm that the following items are present in the figure legend, table legend, main text, or Methods section.

n/a	Confirmed	
☐	☒	The exact sample size (*n*) for each experimental group/condition, given as a discrete number and unit of measurement
☐	☒	A statement on whether measurements were taken from distinct samples or whether the same sample was measured repeatedly
☐	☒	The statistical test(s) used AND whether they are one- or two-sided<br>*Only common tests should be described solely by name; describe more complex techniques in the Methods section.*
☐	☒	A description of all covariates tested
☐	☒	A description of any assumptions or corrections, such as tests of normality and adjustment for multiple comparisons
☐	☒	A full description of the statistical parameters including central tendency (e.g. means) or other basic estimates (e.g. regression coefficient) AND variation (e.g. standard deviation) or associated estimates of uncertainty (e.g. confidence intervals)
☐	☒	For null hypothesis testing, the test statistic (e.g. *F*, *t*, *r*) with confidence intervals, effect sizes, degrees of freedom and *P* value noted<br>*Give P values as exact values whenever suitable.*
☒	☐	For Bayesian analysis, information on the choice of priors and Markov chain Monte Carlo settings
☐	☒	For hierarchical and complex designs, identification of the appropriate level for tests and full reporting of outcomes
☐	☒	Estimates of effect sizes (e.g. Cohen's *d*, Pearson's *r*), indicating how they were calculated

Our web collection on statistics for biologists contains articles on many of the points above.

Software and code

Policy information about availability of computer code

Data collection	Data including metabolites and clinical were collected and stored using Microsoft excel 2003.
Data analysis	Majority of the data analyses were done using R x64 3.4.2. All R codes written for this manuscript are available from the corresponding author upon request.

For manuscripts utilizing custom algorithms or software that are central to the research but not yet described in published literature, software must be made available to editors/reviewers. We strongly encourage code deposition in a community repository (e.g. GitHub). See the Nature Research guidelines for submitting code & software for further information.

Data

Policy information about availability of data

All manuscripts must include a data availability statement. This statement should provide the following information, where applicable:
- Accession codes, unique identifiers, or web links for publicly available datasets
- A list of figures that have associated raw data
- A description of any restrictions on data availability

The data that support the fndings of this study are available from the corresponding author upon reasonable request.

Field-specific reporting

Please select the one below that is the best fit for your research. If you are not sure, read the appropriate sections before making your selection.

☒ Life sciences ☐ Behavioural & social sciences ☐ Ecological, evolutionary & environmental sciences

October 2018

For a reference copy of the document with all sections, see nature.com/documents/nr-reporting-summary-flat.pdf

Life sciences study design

All studies must disclose on these points even when the disclosure is negative.

Sample size	The study was conducted using 2155 patients in four phases. The subjects contain 703 training cohort, 90 validation cohort, 1,248 longitudinal cohort, and 114 drug treatment cohort. One-way ANOVA followed by Dunnett's post hoc test for multiple comparisons was used for three or more groups.
Data exclusions	Patients with acute kidney injury, liver disease, active vasculitis, gastrointestinal pathology or cancer were excluded from the study.
Replication	All attempts at replication were successful.
Randomization	Serum and urine samples were allocated into experimental groups according to their coresponding eGFR value.
Blinding	The investigators were blinded to group allocation during data collection and analysis.

Reporting for specific materials, systems and methods

We require information from authors about some types of materials, experimental systems and methods used in many studies. Here, indicate whether each material, system or method listed is relevant to your study. If you are not sure if a list item applies to your research, read the appropriate section before selecting a response.

Materials & experimental systems

n/a	Involved in the study	
☐	☒	Antibodies
☐	☒	Eukaryotic cell lines
☒	☐	Palaeontology
☐	☒	Animals and other organisms
☐	☒	Human research participants
☒	☐	Clinical data

Methods

n/a	Involved in the study	
☒	☐	ChIP-seq
☒	☐	Flow cytometry
☒	☐	MRI-based neuroimaging

Antibodies

Antibodies used	The following primary antibodies were employed (dilution): collagen I (ab34710, Abcam, USA), α-SMA (ab7817, Abcam, USA), fibronectin (ab2413, Abcam, USA), vimentin (ab92547, Abcam, USA), E-cadherin (ab76055, Abcam, USA), p-IκBα (2859, Cell Signaling Technology, USA), IκBα (4812, Cell Signaling Technology, USA), NF-κB p65 (ab16502, Abcam, USA), MCP-1 (ab7202, Abcam, USA), COX-2 (ab62331, Abcam, USA), Nrf2 (ab31163, Abcam, USA), HO-1 (ab68477, Abcam, USA), NQO-1 (ab28947, Abcam, USA) and TPH-1 (ab52954, Abcam, USA). GAPDH (10494-1-AP) and α-tubulin (11224-1-AP) were purchased from Proteintech Company (Wuhan, China). The secondary antibodies of goat anti-rabbit (ab6721, Abcam, USA), goat anti-mouse (A21010, Abbkine, USA) or rabbit anti-goat (A21110, Abbkine, USA) were used.
Validation	The validation of each primary antibody for the species and application were completely by their producer.

Eukaryotic cell lines

Policy information about cell lines

Cell line source(s)	American Type Culture Collection (ATCC)
Authentication	Human kidney proximal epithelial cells (HK-2) and human mesangial cells (HMC) were authenticated by ATCC.
Mycoplasma contamination	All cell lines have tested negative for mycoplasma contamination.
Commonly misidentified lines (See ICLAC register)	No commonly misidentified cell lines were used in the study .

Animals and other organisms

Policy information about studies involving animals; ARRIVE guidelines recommended for reporting animal research

Laboratory animals	Male BALB/c mice (10 weeks of age), weighing 20–22 g, were used, and the mice were fed according to ARRIVE guidelines.

Wild animals	The study did not involve wild animals.
Field-collected samples	The study did not involve samples collected from the field.
Ethics oversight	This part of animal study was carried out in strict accordance with the recommendations in the Guide for the Care and Use of Laboratory Animals of the State Committee of Science and Technology of the People's Republic of China. The present study has complied with all relevant ethical regulations. All protocols were approved by the Committee on the Ethics of Animal Experiments of the Northwest University (Permit Number: SYXK 2010-004). All surgery was performed under uretane anesthesia, and all efforts were made to minimize suffering. All procedures and care of the rats were in accordance with the institutional guidelines for animal use in research.

Note that full information on the approval of the study protocol must also be provided in the manuscript.

Human research participants

Policy information about studies involving human research participants

Population characteristics	The ages of training cohort are 54±14, 53±16, 52±11, 54±15, 60±15, 58±15 in control and CKD 1-5 stages groups, respectively. The men (%) are 56.8, 49.1, 48.1, 68.1, 52.1 and 47, respectively.
Recruitment	CKD participants were recruited from nephrology department of three hospitals.
Ethics oversight	The part of patient study was approved by the Ethical Committee and all patients provided informed consent prior to entering the study, and all clinical investigation have been conducted according to the principles expressed in the Declaration of Helsinki. The present study has complied with all relevant ethical regulations. The sample collection was approved Shaanxi Traditional Chinese Medicine Hospital (Permit Number: SXSY-235610). For human subjects, written informed consent was received from participants prior to inclusion in the study.

Note that full information on the approval of the study protocol must also be provided in the manuscript.
